# High-Quality Complete Genome Sequences of Three Bovine Shiga Toxin-Producing *Escherichia coli* O177:H- (*fliC*_H25_) Isolates Harboring Virulent *stx2* and Multiple Plasmids

**DOI:** 10.1128/genomeA.01592-17

**Published:** 2018-02-15

**Authors:** Haiqing Sheng, Mingrui Duan, Samuel S. Hunter, Scott A. Minnich, Matthew L. Settles, Daniel D. New, Jennifer R. Chase, Matthew W. Fagnan, Carolyn J. Hovde

**Affiliations:** a Bi-State School of Food Science, University of Idaho, Moscow, Idaho, USA; b Institute for Bioinformatics and Evolutionary Studies (IBEST), University of Idaho, Moscow, Idaho, USA; c Bioinformatics Core, University of California, Davis, Davis, California, USA; d Department of Biology, Northwest Nazarene University, Nampa, Idaho, USA

## Abstract

Shiga toxin-producing *Escherichia coli* (STEC) bacteria are zoonotic pathogens. We report here the high-quality complete genome sequences of three STEC O177:H- (*fliC*_H25_) strains, SMN152SH1, SMN013SH2, and SMN197SH3. The assembled genomes consisted of one optical map-verified circular chromosome for each strain, plus two plasmids for SMN013SH2 and three plasmids for SMN152SH1 and SMN197SH3, respectively.

## GENOME ANNOUNCEMENT

Shiga toxin (Stx)-producing *Escherichia coli* (STEC) can cause hemorrhagic colitis and hemolytic uremic syndrome (1–3). Cattle are major reservoirs of STEC and are the most common source for foodborne infections (4–6). During a longitudinal survey of STEC in healthy cattle, we isolated three non-O157 STEC strains that share phenotypic similarity with the O157 serogroup (7). Because these strains were nonmotile when isolated, DNA microarray for STEC serotyping (8) confirmed that these strains (SMN152SH1, SMN013SH2, and SMN197SH3, formerly named SH1, SH2 and SH3 respectively.) belonged to serogroup O177:H25. Based on these data we reassigned the serotype of these strains to O177:H- (*fliC*_H25_: *fliC* genotype H25). Among the surveyed cattle in Washington and Idaho, US, and Alberta, Canada, the O177:H- serotype was more prevalent than the O157:H7 serotype (7). We report here the availability of three high-quality complete genomes of STEC O177:H- assembled using a hybrid approach that combines PacBio and Illumina reads.

Genomic DNA was prepared using a GenElute bacterial Genomic DNA kit (Sigma-Aldrich, St. Louis, MO) per the manufacturer’s instructions. Whole-genome sequencing was carried out on a PacBio RS II (Pacific Biosciences, Menlo Park, CA) platform with 6-kb and 20-kb insert libraries by using P6 polymerase-binding and C4-sequencing kits for 240-min acquisition. Short-read sequencing was done at the IBEST Genomics Resources Core at the University of Idaho, using an Illumina MiSeq and v2 500-cyles kit (PE250) following construction of barcoded Illumina libraries using the IntegenX Apollo 324 PrepX ILM DNA library kit and custom Illumina TruSeq barcoded adapters. The Illumina reads were cleaned using HTStream (https://github.com/ibest/HTStream) to trim adapter sequences and low-quality ends and to overlap paired-end reads. Long reads were assembled with Canu v1.6 using default parameters (9). Gaps between contigs were sealed with GapBlaster (10). The small plasmids of SMN152SH1 and SMN197SH3 were assembled with Unicycler (11). The assembled genomes were oriented and trimmed using Circlator v1.5.2 (12), then corrected with Pilon v1.22 using cleaned Illumina reads (13). The resulting assemblies were verified using restriction enzyme NcoI whole-genome maps generated according to the OpGen protocol (OpGen, Inc., Gaithersburg, MD).

The genomes were annotated using the NCBI Prokaryotic Genome Annotation Pipeline (https://www.ncbi.nlm.nih.gov/genome/annotation_prok/). Features of the three O177:H- isolates include an *stx*_2c_ variant gene which is 100% identical to the *stx*_2c_ carried by a clinical isolate *E. coli* O177 (14); nearly identical pO157-like 88-kb and 48-kb plasmids, a 6.7-kb colicinogenic plasmid in SMN152SH1 and SMN197SH3; and chromosomally encoded AmpC type β-lactamase.

A detailed report on additional analysis of these genomes will be included in a future publication.

### Accession number(s).

Accession numbers and assembly metrics for each complete genome sequence are listed in Table 1.

**TABLE 1:** Accession numbers and assembly metrics of the three annotated STEC O177:H- genomes

Strain	Plasmid	Accession no.	Genome size (bp)	GC content (%)	Plasmid size (bp)
SMN152SH1		CP024618	5,302,502	50.7	
	pO177A1	CP024617			88,896
	pO177B1	CP024616			48,983
	pO177C1	CP024615			6,675
SMN013SH2		CP023673	5,143,533	50.5	
	pO177A2	CP023674			87,524
	pO177B2	CP023675			46,664
SMN197SH3		CP024056	5,267,207	50.7	
	pO177A3	CP024055			88,839
	pO177B3	CP024054			48,982
	pO177C3	CP024053			6,675
